# Circulating angiopoietin-like 8 (ANGPTL8) is a marker of liver steatosis and is negatively regulated by Prader-Willi Syndrome

**DOI:** 10.1038/s41598-017-03538-7

**Published:** 2017-06-09

**Authors:** Chiara Mele, Graziano Grugni, Stefania Mai, Roberta Vietti, Gianluca Aimaretti, Massimo Scacchi, Paolo Marzullo

**Affiliations:** 1 Division of General Medicine, Ospedale S. Giuseppe, Istituto Auxologico Italiano, via Cadorna 90, 28824 Piancavallo di Oggebbio (VB), Italy; 2 Division of Auxology, Ospedale S. Giuseppe, Istituto Auxologico Italiano, via Cadorna 90, 28824 Piancavallo di Oggebbio (VB), Italy; 3 Laboratory of Metabolic Research, Ospedale S. Giuseppe, Istituto Auxologico Italiano, via Cadorna 90, 28824 Piancavallo di Oggebbio (VB), Italy; 4 Department of Translational Medicine, University of Piemonte Orientale, via Solaroli 17, 28100 Novara, Italy

## Abstract

ANGPTL8 is a liver-derived protein related to insulin-sensitivity. Its relationship with obesity and liver function in Prader-Willi syndrome (PWS) is unknown. The present study investigated circulating ANGPTL8 in PWS and controls with common obesity, assessing its association to liver steatosis. For this purpose, 20 obese PWS and 20 controls matched for body mass index (BMI), sex and age underwent analysis of ANGPTL8 levels, glucose and lipid metabolism. Liver function tests and degree of liver steatosis by ultrasonography (US), fat-free mass (FFM) and fat mass (FM) by dual-energy x-ray absorptiometry (DEXA) were also assessed. In comparison to controls, obese PWS showed lower values of FFM (p < 0.0001) and higher FM (p = 0.01), while harbouring higher HDL cholesterol, lower triglycerides and OGTT-derived insulin levels, as well as a lower prevalence and severity of liver steatosis. With respect to obese controls, ANGPTL8 levels were significantly lower in PWS (p = 0.007) and overall correlated with transaminase levels and the severity of liver steatosis, as well as FFM (p < 0.05 for all). By a stepwise multivariable regression analysis, ANGPTL8 levels were independently predicted by PWS status (p = 0.01) and liver steatosis (p < 0.05). In conclusion, ANGPTL8 levels are lower in PWS than obese controls and are inversely associated with the severity of liver steatosis. Further studies should investigate the potential genetic basis for this observation.

## Introduction

Prader-Willi syndrome (PWS) is an imprinted neurobehavioral condition caused by the lack of expression of genes located on the paternal chromosome 15q11.2-q13. There are three main genetic subtypes in PWS: paternal 15q11-q13 deletion (65–75% of cases), maternal uniparental disomy of chromosome 15 (UPD15) (20–30% of cases), and imprinting defects (1–3%)^1^. The smallest deletions discovered to date demonstrate that the SNORD116 snoRNA gene cluster can explain much of the PWS phenotype^2^. Clinically, PWS is characterized by neonatal hypotonia and failure to thrive, cognitive and behavioural disorders, endocrine defects such as short stature and hypogonadism, autonomic dysregulation. PWS is tipically associated with a lack of satiety, which generates obsessive craving for food and results in extreme obesity by the adult age^3^.

Adult patients with PWS show peculiar body and metabolic features. Compared to BMI-matched controls, PWS patients harbour a predominant accumulation of subcutaneous adipose tissue, with lower accumulation of visceral adipose tissue than that observed in patients with common obesity^4–6^. In addition, lean body mass and muscle function is impaired^7^, and results in reduced resting energy expenditure (REE) and decreased voluntary activity^8^. Despite this unfavourable body composition, the metabolic phenotype of PWS is characterized by lower insulin levels and higher insulin sensitivity as opposed to obese controls^9, 10^, while dyslipidaemia rarely occurs in PWS^11^. Although the molecular mechanisms driving this peculiar metabolic profile are not well understood, it can be hypothesized that the elevation of orexigenic hormones, such as ghrelin^12, 13^, and different expression of adipocytokines^14^, particularly adiponectin^15^, could intervene to regulate the metabolic profile of PWS adults^16^.

A recently identified liver protein, angiopoietin-like 8 (ANGPTL8), has been described to be involved in different metabolic pathways related to glucose and lipid metabolism^17–19^. As summarized by Tseng *et al*., several researchers have investigated the subcellular localization of ANGPTL8, demonstrating that its cytoplasmic vesicle-like distribution is likely involved in the lipid regulation pathway^17^. Serum ANGPTL8 has been detected in sera from humans and mice, with its levels found to be positively correlated with triglycerides (TG) and very low-density lipoprotein (VLDL) levels^18^. Intracellular ANGPTL8 is associated with lipid droplets, suggesting that ANGPTL8 may serve as a lipoprotein and could be secreted or taken up with a lipid-associated compartment^18^.

Regarding its function/s in glucose homeostasis, ANGPTL8 might be key in regulating postprandial glucose metabolism. In fact, liver ANGPTL8 over-expression in mice increases insulin-mediated synthesis of glycogen^19^. Moreover, it is able to promote the suppression of key enzymes involved in gluconeogenic pathways, thereby improving insulin resistance^19^.

To date, the metabolic significance of ANGPTL8 in human obesity and obese patients with PWS is unknown. As such, this study was designed to investigate the relationship between ANGPTL8 levels and adiposity, metabolic homeostasis and liver steatosis in association with obesity and the PWS condition.

## Methods

### Patients

This study enrolled 40 patients, consisting of 20 PWS adults with obesity (10 F/10 M; age, 34.2 ± 7.6 years; BMI, 45.5 ± 9.4 kg/m^2^) and 20 BMI-matched control subjects (10 F/10 M; age, 35.0 ± 8.3 years; BMI, 48.6 ± 10.2 kg/m^2^), referred to our institution for work-up and rehabilitation of obesity and its comorbidities. All PWS individuals received a diagnosis based on typical syndromic features confirmed by molecular genetic studies of chromosome 15, including 15q11-q13 deletion in 16 (10 males and 6 females) and UPD15 in the remaining 4 females. Exclusion criteria included any liver disease except for newly diagnosed steatosis, kidney failure, autoimmune diseases, uncontrolled hypothyroidism and/or diabetes mellitus, exposure to glucocorticoids or alcohol consumption. With respect to hormone replacement therapy in PWS, 9 patients were treated with rhGH, 2 female patients with estrogens and 2 patients with levothyroxine. Four PWS patients were treated with psychotropic medications. The experimental procedure was approved by the ad hoc Ethical Research Committee of the Istituto Auxologico Italiano, Verbania, Italy. A written informed consent was obtained from the PWS patients and their parents or guardians, and from the obese participants. The study protocol conformed to the guidelines of the European Convention on Human Rights and Biomedicine concerning biomedical research.

### Body measurements and instrumental tests

All subjects underwent body measurements wearing light underwear, in fasting conditions after voiding. Weight and height were measured to the nearest 0.1 kg and 0.1 cm, respectively, using standard methods. BMI was expressed as body mass (kg)/height (m)^2^. Obesity was defined for any BMI over 30 kg/m^2^ ^20^. Waist circumference (WC) was measured midway between the lowest rib and the top of the iliac crest after gentle expiration; hip measurements were taken at the greatest circumference around the nates.

A dual-energy x-ray absorptiometry (DEXA; GE Lunar, Madison, WI, USA) was performed for the assessment of body mass. This was expressed as lean body mass in kilograms and fat body mass as the percentage of total body mass.

The REE was expressed in kilocalories/24 h and determined in a thermoregulated room (22–24 °C) by computed open-circuit indirect calorimetry, measuring resting oxygen uptake and resting carbon dioxide production by a ventilated canopy (Sensormedics, Milan, Italy) at 1-min intervals for 30 min, expressed as 24 h value.

In order to assess the presence and severity of liver steatosis, liver US was performed by the same operator who was blinded to the laboratory and clinical data at the time of the procedure, using a high-resolution US system (LOGIQ 7, GE Healthcare, Waukesha, WI, USA). The degree of hepatic steatosis was assessed semi-quantitatively on the basis of hepatorenal echo contrast, liver brightness, deep attenuation and vascular blurring. Liver steatosis was established by a validated method of US grading (categorized as: G0 = absent; G1 = mild; G2 = moderate, G3 = severe steatosis)^21^, to accomplish for the subjective difficulties of PWS patients to undergo invasive or radiological assessment (MRI) where patients’ collaboration was needed.

### Laboratory tests

Blood samples were drawn under fasting conditions, centrifuged, and stored at −80 °C until required.

Serum ANGPTL8 levels were assessed using a commercially available human EIA kit (Phoenix Pharmaceutics, Inc, Burlingame, CA, USA). The assay procedure was performed in accordance with the manufacturer’s instructions. All samples were analyzed in duplicate. Intra-assay CV and inter-assay CV of ANGPTL8 were less than 10% and 15% respectively. Minimum detectable concentration was 0.12 ng/mL. Furthermore, the EIA was specific for human ANGPTL8. In addition, quality controls were included in all EIA measurements with the results within the expected range.

Routine laboratory data included levels of C-reactive protein, alkaline phosphatase, aspartate aminotransferase (AST), alanine aminotransferase (ALT), gamma-glutamyl transpeptidase (GGT), glucose, total cholesterol, high-density (HDL) and low-density lipoprotein (LDL) cholesterol, triglycerides (TG) and glycated haemoglobin (HbA1c), measured by enzymatic methods (Roche Diagnostics, Mannheim, Germany). Levels of insulin were measured using a Cobas Integra 800 Autoanalyzer (Roche Diagnostics, Indianapolis, IN, USA). Ultrasensitive C-reactive protein (CRP) was measured by CRP (latex) HS Roche kit.

Glucose homeostasis was evaluated by oral glucose tolerance test (OGTT) in all subjects and glucose tolerance was expressed, according to ADA recommendations^22^, as normal fasting plasma glucose (FPG) if <100 mg/dl (5.6 mmol/l); impaired FPG (IFG) if FPG was 100–125 mg/dl (6.9 mmol/l); impaired glucose tolerance (IGT) if 2-h post-OGTT plasma glucose was 140–199 mg/dl (7.8–11.0 mmol/l); T2DM if FPG was ≥126 mg/dl (≥7 mmol/l) two days apart, or if 2-h post-OGTT plasma glucose was ≥200 mg/dl (≥11.1 mmol/l). HbA1c values of 5.7 and 6.5% were considered as the threshold of normal glucose metabolism and T2DM, respectively.

Matsuda^23, 24^ and Stumvoll^25^ indexes were used to measure insulin sensitivity obtained from plasma glucose and insulin concentrations during the oral glucose tolerance test (OGTT). The Matsuda index provides a reasonable index of whole-body insulin sensitivity^23, 24^ and was calculated using the following formula: 10,000/square root of [(fasting glucose × fasting insulin) × (glucose × insulin at time 120 min during OGTT)]. The Stumvoll index is a predictor of individual’s insulin sensitivity and β-cell function^25^ and was calculated using the following formula: 0.156 − (0.0000459 × insulin at time 120 min during OGTT) − (0.000321 × fasting insulin) − (0.00541 × glucose at time 120 min during OGTT).

Insulin resistance was calculated by the homeostatic model of insulin resistance (HOMA-IR) index: insulin (mIU/L) × [glucose (mmol/L)/22.5]^26^. A HOMA-IR value greater than 2.0 was considered indicative of insulin resistance, as obtained in a sample of the Italian population^27^. The homeostatic model of β cell function (HOMA-B) was used to describe the functionality of pancreatic beta cells and calculated using the following formula: 20 × [insulin (mIU/L)/glucose (mmol/L) − 3.5]^26^.

### Data analysis

Statistical analysis was performed using SPSS version 18 (Somers, NY, USA). Values are expressed as means ± standard deviation (SD). For comparative analysis, ANOVA between the 2 groups and paired-T test intra-groups were used. For comparative analysis of ANGPTL8 levels across liver steatosis categories, Kruskall-Wallis test with Dunn’s correction was used. Pearson’s correlation analysis and the Chi square were used to identify significant associations between variables of interest. Stepwise multivariate regression analysis was used to evaluate the independent association of variations in ANGPTL8 with metabolic, anthropometric or biochemical parameters. The multilinear model included PWS status (no PWS = 0; PWS = 1), liver steatosis or ALT levels, age, gender and BMI as independent variables. β coefficients and related significance values obtained from the models are reported. P < 0.05 was considered as statistically significant.

## Results

**Figure 1:**
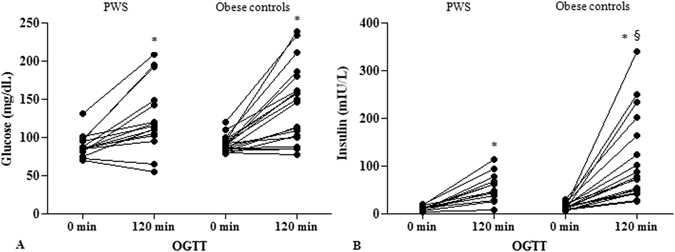
OGTT-derived glucose levels (mg/dl) (**A**) and insulin levels (mIU/L) (**B**) obtained at time 0 min and 120 min in PWS and controls. Intra-groups differences were assessed by paired T-test. Inter-group analyses were performed by ANOVA. For significance: *p < 0.0001 by paired T-test; ^§^p = 0.02 by ANOVA.

A summary of anthropometric and biochemical data is reported in Tables 1 and 2, and Fig. 1. BMI values were comparable between the two groups and ranged, collectively, between 31.5–73.3 kg/m^2^. Severe obesity (BMI > 40 kg/m^2^) was present in 67.5% of cases (13 PWS and 14 obese patients). Abnormal glucose metabolism was detected in 50% of PWS and 55% of obese controls (T2DM in 25% of PWS and 20% of obese subjects, IGT in 20% of PWS and 35% of obese controls, IFG in 5% of PWS and no controls).

**Table 1:** Summary of anthropometric data obtained in PWS subjects and obese controls.

Variables	PWS (n = 20)	Obese (n = 20)	P Value
Males/females	10/10	10/10	—
Age (years)	34.2 ± 7.6	35.0 ± 8.3	0.7
BMI (kg/m^2^)	45.5 ± 9.4	48.6 ± 10.2	0.3
Weight (Kg)	**105.9** ± **23.5**	**137.6** ± **27.2**	**<0.001**
Height (cm)	**152.6** ± **8.1**	**168.5** ± **10.5**	**<0.0001**
Waist (cm)	124.8 ± 15.2	132.6 ± 12.8	0.09
Hip (cm)	130.1 ± 16.8	140.4 ± 18.8	0.08
Waist-to-hip ratio	0.96 ± 0.08	0.95 ± 0.11	0.7
FM (%)	**53.7** ± **6.1**	**47.5** ± **8.1**	**0.01**
FFM (Kg)	**47.8** ± **11.6**	**70.2** ± **13.7**	**<0.0001**
REE (kcal/day)	**1607.5** ± **281.3**	**2198.6** ± **402.1**	**<0.0001**

Data are expressed as mean ± SD. Comparison between populations was performed by ANOVA test. Significant differences are shown in bold characters.

BMI, body mass index; FM, fat mass; FFM, fat free mass; REE, resting energy expenditure.

**Table 2:** Summary of biochemical data obtained in PWS subjects and obese controls.

Variables	PWS (n = 20)	Obese (n = 20)	P Value
ANGPTL8 (ng/mL)	**0.58** ± **0.21**	**0.93** ± **0.50**	**0.007**
Glucose OGTT_0_ (mg/dL)	100.6 ± 31.6	90.6 ± 10.0	0.1
Glucose OGTT_120_ (mg/dL)	125.1 ± 44.9	142.2 ± 47.8	0.3
Insulin OGTT_0_ (mIU/L)	10.7 ± 5.0	13.5 ± 6.2	0.1
Insulin OGTT_120_ (mIU/L)	**53.4** ± **28.1**	**106.6** ± **86.5**	**0.02**
C- Peptide (μg/L)	**2.3** ± **0.9**	**3.2** ± **0.9**	**0.002**
Matsuda	6.39 ± 6.37	3.51 ± 2.25	0.06
Stumvoll	**0.077** ± **0.029**	**0.051** ± **0.045**	**0.05**
HOMA-IR	2.6 ± 1.6	3.0 ± 1.5	0.4
HOMA-B	149.4 ± 57.0	194.9 ± 109.3	0.1
HbA1c (%)	5.8 ± 1.0	5.7 ± 0.5	0.5
AST (U/L)	**18.9** ± **6.7**	**29.1** ± **15.1**	**0.01**
ALT (U/L)	**25.0** ± **16.9**	**40.5** ± **29.9**	**0.02**
GGT (U/L)	36.9 ± 50.0	34.0 ± 26.6	0.8
ALP (U/L)	77.1 ± 19.6	73.7 ± 14.9	0.5
CHO (mg/dL)	178.6 ± 42.6	186.9 ± 25.2	0.4
LDL CHO (mg/dL)	118.9 ± 39.3	120.4 ± 21.9	0.8
HDL CHO (mg/dL)	**48.9** ± **11.1**	**41.1** ± **9.2**	**0.02**
TG (mg/dL)	**97.3** ± **35.1**	**131.6** ± **45.1**	**0.01**
Urate (mg/dL)	**5.4** ± **1.0**	**6.5** ± **1.1**	**0.004**
CRP (mg/dL)	1.2 ± 1.3	1.1 ± 0.7	0.7

Data are expressed as mean ± SD. Comparison between populations was performed by ANOVA test. Significance is shown in bold characters.

OGTT, Oral Glucose Tolerance Test; OGTT_0_ and OGTT_120_, OGTT at time 0 min and 120 min; HOMA-IR, homeostatic model of insulin resistance; HOMA-B, homeostatic model of β cell function; HbA1c, glycated haemoglobin; AST, aspartate aminotransferase; ALT, alanine aminotransferase; GGT, gamma glutamyl transpeptidase; ALP, alkaline phosphatase; CHO, total cholesterol; LDL CHO, low density lipoprotein cholesterol; HDL CHO, high density lipoprotein cholesterol; TG, triglycerides; CRP, C-reactive protein.

Anthropometric parameters differed between groups. PWS showed lower values of REE (p < 0.0001) and FFM (p < 0.0001), and higher %FM (p = 0.01) with respect to obese controls. Nevertheless, in the PWS group we observed lower OGTT-derived insulin (p = 0.02), higher HDL (p = 0.02 for all) and lower TG (p = 0.01) and urate levels (p = 0.004), which was suggestive of a better metabolic profile in PWS compared to obese controls.

Liver function tests and liver steatosis evaluated by US resulted less impaired in PWS than obese controls. In fact, transaminases levels were high (>40 U/L) in 3 PWS and in 7 obese patients (15% vs. 35%), while US documented liver steatosis in 14 PWS and 18 obese controls (70% vs. 90%; χ^2^ = 12.5, p < 0.001). According to the grading of liver steatosis, PWS had a higher prevalence of G0 (30% vs. 7%; χ^2^ = 17.5, p < 0.0001), a similar prevalence of G1 (20% vs. 20%, respectively) and G2 (30% vs. 33%, respectively), and a lower prevalence of G3 score (20% vs. 40%; χ^2^ = 9.5, p < 0.01), when compared with obese controls.

**Figure 2:**
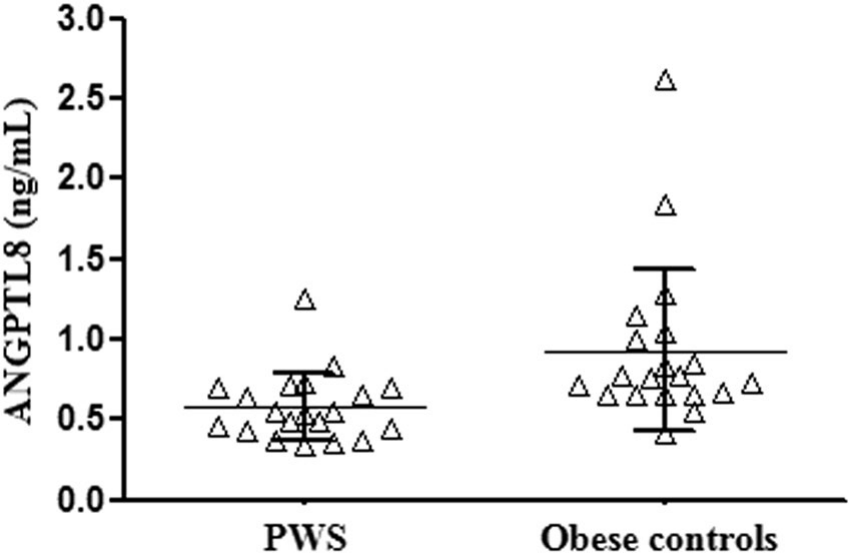
Individual values of circulating ANGPTL8 levels obtained in PWS patients and obese controls. Lines represent mean and standard deviation values in the two populations.

**Figure 3:**
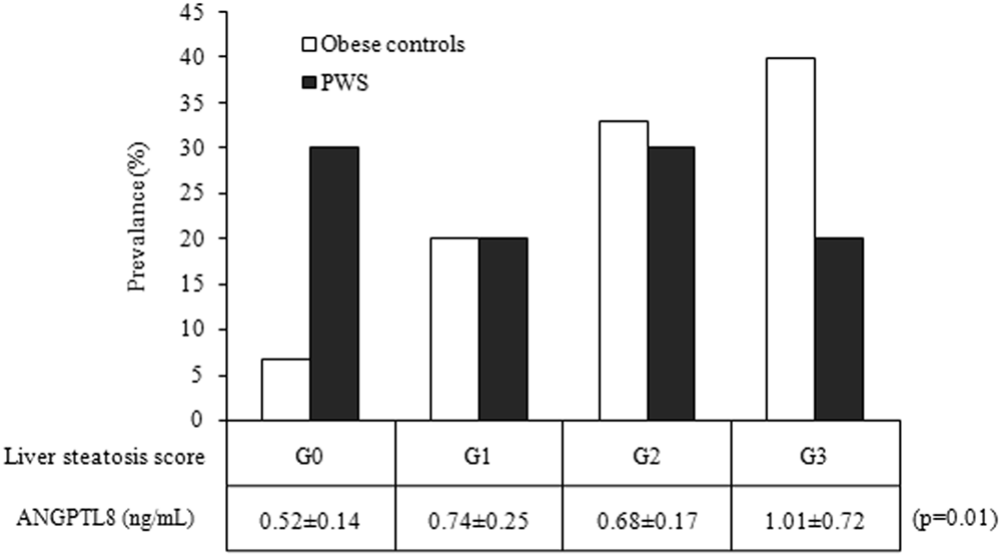
Histogram illustrating the prevalence (%) of liver steatosis in PWS and obese controls, and the corresponding ANGPTL8 levels across liver steatosis scores as obtained in the two population as a whole. Significance is expressed as obtained by ANOVA. Inter-group differences are listed in the Results section.

Analysis of circulating ANGPTL8 showed overall significantly lower levels in PWS compared to obese controls (Fig. 2). ANGPTL8 levels were only modestly overlapping between populations and were less variable in PWS than obese subjects. The greater dispersion around the mean of ANGPTL8 values in the obese group likely reflected differences in liver function, as confirmed by an elevation of AST and ALT levels in both outliers of the obese group of controls (Fig. 2). Corroborating these findings, correlation analysis in the two groups as a whole (Table 3) showed significant positive correlations between ANGPTL8 and AST (r = 0.35, p < 0.05), ALT (r = 0.38, p = 0.01), as well as with the grading of liver steatosis (r = 0.36, p < 0.05). These relationships were further substantiated by the observation of parallel increases of ANGPTL8 levels with the severity of liver steatosis (p = 0.01 by repeated measures ANOVA) (Fig. 3), and by association observed between ANGPTL8 levels and liver steatosis in the group of obese controls (r = 0.53, p < 0.05). Of note, a significant association was also seen between ANGPTL8 levels and REE (r = 0.32, p = 0.05) as well as FFM (r = 0.31, p < 0.05), while only in PWS ANGPTL8 levels were inversely associated with %FM (r = −0.46, p < 0.05). Significant correlations obtained on the entire dataset were lost after controlling for PWS status, thereby confirming the blunting effect of PWS on ANGPTL8 levels (r = −0.42, p < 0.01). There were no differences in ANGPTL8 levels and liver steatosis when PWS group was analysed according to GH treatment or genotype (data not shown).

**Table 3:** Pearson’s correlation analysis between ANGPTL8 levels and anthropometric and biochemical parameters in the study populations as a whole.

Parameters	ANGPTL8 levels	
	r	p-value
Age (years)	0.04	0.7
Status	**−0.42**	**0.007**
BMI (kg/m^2^)	−0.04	0.7
FM (%)	−0.26	0.1
FFM (kg)	**0.31**	**0.04**
REE (kcal/day)	**0.32**	**0.05**
Glucose OGTT_0_ (mg/dL)	−0.10	0.5
Glucose OGTT_120_ (mg/dL)	0.30	0.08
Insulin OGTT_0_ (mIU/L)	0.09	0.5
Insulin OGTT_120_ (mIU/L)	0.30	0.08
C- Peptide (μg/L)	0.30	0.07
AST (U/L)	**0.35**	**0.02**
ALT (U/L)	**0.38**	**0.01**
Liver steatosis score	**0.35**	**0.03**

For obese status: PWS = 1, common obese = 0. Significance is shown in bold characters.

BMI, body mass index; FM, fat mass; FFM, fat free mass; REE, resting energy expenditure; OGTT, Oral Glucose Tolerance Test; OGTT_0_ and OGTT_120_, OGTT at time 0 min and 120 min; AST, aspartate aminotransferase; ALT, alanine aminotransferase.

Stepwise multivariable regression analysis documented that ANGPTL8 levels were negatively predicted by PWS status (standardized β = −0.41, p = 0.01). After the removal of PWS status from the regression equation, ALT levels (standardized β = 0.39, p = 0.01) or the score of liver steatosis (standardized β = 0.35, p < 0.05), acted as independent predictors of ANGPTL8 levels.

## Discussion

The present study analysed the association between ANGPTL8 levels and adiposity, metabolic profile and liver steatosis in relation to the adult PWS condition and obesity. Our results show that PWS patients harbor lower ANGPTL8 levels than obese controls and that ANGPTL8 levels are more closely associated with liver steatosis, than with body composition and metabolic homeostasis.

The metabolic phenotype of PWS is different as compared to common obesity and some metabolic complications typically related to obesity, such as insulin resistance and reduced hepatic insulin extraction, are less severe than expected for the degree of fat accumulation^28^. In addition, PWS is associated with peripheral rather than central distribution of body fat^4^, less severe metabolic signatures in adipocytes^16^, and abnormalities in growth hormone secretion, ghrelin levels and adipokine patterns, when compared to common obesity^12, 14–16^. From a clinical viewpoint, factors influencing these discrepancies are incompletely understood.

ANGPTL8 is a liver and adipose tissue-produced protein, involved in the regulation of triglyceride and glucose metabolism. Its activity involves the ability of reducing serum triglyceride clearance and improving insulin resistance^17, 29–33^. In the current study, we observed lower circulating levels and less interindividual variability of ANGPTL8 in PWS when compared to subjects with common obesity. While insulin resistance and whole-body insulin sensitivity index did not greatly differ between PWS and obese controls, several anthropometric and metabolic differences existed between the two populations. Particularly, PWS patients showed higher HDL cholesterol and lower TG levels than their control counterpart. Previous studies have suggested that PWS is characterized by a more efficient triglyceride storage likely due to an increase of adipose tissue lipoprotein lipase (LPL) activity, suggesting an altered pathway of fat mobilization and oxidation^34^. Noteworthy, ANGPTL8 shows the ability to suppress triglyceride clearance through the inhibition of LPL, thus increasing serum triglycerides^33^. Therefore, we speculate that the decreased serum ANGPTL8 levels in PWS could contribute in explaining the lower triglyceride levels seen in PWS when compared to obese controls. In our study groups, the role of circulating ANGPTL8 on glucose homeostasis and insulin resistance appeared to be of little relevance compared to data in the literature^19^. In fact, no correlations between ANGPTL8 and glucometabolic parameters were observed. These results do not disagree with recent data, but rather suggest that ANGPTL8 is not as robustly involved in β-cell proliferation as originally proposed^35^, at least in our study populations.

In the search for mechanisms to explain our observations, we noted that circulating ANGPTL8 paralleled the behaviour of crucial determinants of metabolic health, such as liver steatosis. In fact, circulating ANGPTL8 levels were negatively associated with indices of liver steatosis, i.e. transaminases and US-derived scores of steatosis, which are recognized non-invasive markers of liver impairment in obesity^36–38^. While liver biopsy is the gold-standard method for the accurate staging of non-alcoholic liver steatosis, several previous studies demonstrated a strong correlation between US findings and the degree of liver steatosis documented by biopsy^21, 39, 40^. Bearing in mind the limitation of our approach, current data confirm that liver steatosis is less severe in PWS than in common obesity^37^, and substantiates the emerging role of liver steatosis on circulating ANGPTL8 in severe obesity, which is further strengthened by the results of correlation analyses and multivariable regression analyses. Being a primarily liver-produced protein, ANGPTL8 is positively associated with biochemical indices of liver injury and steatosis in overweight and obese individuals^41^. In hepatoma cells, Tseng YH *et al*. demonstrated that ANGPTL8 is mainly localized in the cytoplasm with a vesicle-like distribution^18^, possibly implying that hepatocyte lysis linked to steatosis could promote the leakage of ANGPTL8 vesicle in the bloodstream, thus helping to explain our findings. Complementing literature data^41, 42^, our results suggest that ANGPTL8 levels could act as a novel surrogate biomarker for liver steatosis in non-PWS obese individuals. As certain adipokines can predict the severity of liver steatosis^43^, however, studies are required to clarify the association between adipocytokines and ANGPTL8 levels in steatosis.

In conclusion, ANGPTL8 levels are lower in PWS than obese controls and, overall, they seem to reflect the severity of liver steatosis. Further studies should investigate the potential genetic basis of these findings.
